# MEK inhibitors in non-V600 BRAF mutations and fusions

**DOI:** 10.18632/oncotarget.27788

**Published:** 2020-11-03

**Authors:** Douglas B. Johnson, Caroline A. Nebhan, Marcus S. Noel

**Affiliations:** ^1^Department of Medicine, Vanderbilt University Medical Center and Vanderbilt Ingram Cancer Center, Nashville, TN, USA; ^2^Department of Medicine, Georgetown University Medical Center, Washington, DC, USA

**Keywords:** trametinib, MEK, BRAF, atypical, fusion

## Abstract

Mutations in BRAF at the 600th codon have proven sensitive to combination BRAF and MEK inhibition. Mutations outside this codon, however, are approximately as common but do not have approved targeted therapy approaches. Herein, we discuss targeting these non-V600 mutation and fusions in BRAF with MEK inhibitors.

## INTRODUCTION

Developing active, molecularly targeted agents against mutated *BRAF* has been a major success story of the precision-cancer movement. Mutations affecting the 600th codon may be targeted by several approaches, including mutant-specific BRAF inhibitors, blockers of downstream signal partners MEK1/2, or the combination of both. In particular, the management of *BRAF* V600-mutant melanoma, lung cancer, hairy cell leukemia, and thyroid cancer has been transformed by these agents, which produce high response rates, occasionally resulting in durable responses [1–5].

In contrast to the high-profile success of pharmacologic blockade of mutant *BRAF* V600, approaches to targeting other mutations in *BRAF* outside of the 600th codon have been less active. When taken together, these mutations include over 200 BRAF-mutant alleles corresponding to about 30 distinct BRAF mutations. Non-V600 mutations are approximately as common as V600 mutations, and are found in 1–3% of all cancers, including in many common histology types (lung, colon, prostate, gynecologic malignancies) as well as less common tumor types (primary brain tumors, neuroendocrine tumors, and hematologic malignancies) [6–8]. These mutations all seem to activate mitogen activated protein kinase (MAPK) pathway signaling and may be classified into one of three different types based on their mechanism of action [9–11]. Class 1 mutations, which are exclusively *BRAF* V600 mutation, signal as monomers in constitutively active fashion. Class 2 mutations function as constitutively active dimers. Class 2 mutations include L597 and K601 mutations, which have been subject to several case reports demonstrating activity for MEK inhibition. Class 3 mutations, which include G466, D594, and A581 mutations, induce preferential binding to wild type RAF, and are kinase dead or have impaired kinase activity. These mutations, however, are still associated with enhanced RAS/MAPK activation, potentially due to other mechanisms such as growth factor signaling or concurrent MAPK pathway mutations (e.g., *RAS* or *NF1*) [10].

Several pre-clinical studies and isolated case reports have demonstrated impressive efficacy for MEK inhibitors in patients with non-V600 *BRAF* mutations, as well as *BRAF* fusions [12–16]. These studies, however, were largely conducted in melanoma patients (a histology where MEK inhibitors are approved therapy, and have activity in *BRAF* V600 mutant melanoma), and with class 2 mutations (specifically for L597 and K601 mutations) or fusions. Thus, a larger study to test the concept more generally was planned.

With that backdrop, we studied trametinib, a second-generation allosteric inhibitor of MEK1/2 in patients with solid tumors and lymphomas harboring mutations in *BRAF* outside the V600 codon, as well as BRAF fusions [17]. This subprotocol was a subset of the National Cancer Institute Molecular Analysis for Therapy Choice (NCI-MATCH) study, a large platform trial with multiple parallel phase II studies that were designed to evaluate genomically-targeted therapies matched to specific genomic alterations. Per study design, 32 patients with non-V600 *BRAF* alterations (13 class 2 mutations, 19 class 3 mutations, and 1 fusion) were enrolled, and received trametinib 2 mg daily until disease progression (dose reduction permitted for severe toxicity). Patients were heavily pre-treated (69% with 3 or more lines of therapy), and most often had cancers of the lung (*n* = 9), colon/rectum (*n* = 7), and prostate (*n* = 4). Unfortunately, activity was disappointing. Of these, one patient with breast cancer and a *BRAF* G469E mutation (class 2) had a partial response (response rate 3%), one patient with lung adenocarcinoma and a *BRAF* G469A mutation remains on therapy at 20.4 months, and an additional 3 patients had progression-free survival (PFS) of > 6 months. Median PFS and overall survival were 1.8 and 5.7 months, respectively.

Why the lack of activity? We did attempt to identify explanations within the trial data, although no subgroups had exceptionally good outcomes. Trends toward worse outcomes were noted in patients with colorectal cancer, high *BRAF* mutant allele frequency, and class 3 mutations, whereas concurrent mutations (including *RAS* mutations) did not impact outcomes. We speculate that histology does still matter, as many patients in the study had tumors that have historically been resistant to MAPK pathway inhibition (e.g., colon cancer) [18, 19], and only 1 patient had melanoma. It is possible that tumors with class 3 mutations harbored many alternative genetic and epigenetic causes of alternative cellular pathway activation, such that simply blocking MEK1/2 was not sufficient for growth attenuation [10]. Exceedingly low incidence of recurrent BRAF fusions limited enrollment of such patients (1 patient), though at least some prior case reports suggest potential for good response with MEK inhibition [13, 20, 21].

What are the next steps? First, our study does not change available data suggesting that MEK inhibition may be still considered for melanoma patients whose tumors harbor class 2 *BRAF* mutations [22, 23], or perhaps non-small cell lung cancer patients [24]. More recent studies have even suggested that combining BRAF and MEK inhibitors is the most active strategy in these patients [14, 25]. However, this is only a modestly effective solution for a small subset of these patients, and MEK inhibition cannot be recommended for most other patients with non-V600 *BRAF* mutations. Second, one approach might be studying dual blockade of MAPK pathway and other parallel signaling networks (e.g., PI3K pathway). These types of approaches, however, have neither promising activity nor safety in early studies [26–28]. However, histology specific approaches, such as combination of BRAF, MEK, and EGFR inhibition in *BRAF* V600-mutant colon cancer demonstrate, may hold promise as well [29]. Third, more complete extinguishment of MAPK signaling may hold promise. Early studies have shown that blocking the final member of the pathway, ERK, may produce responses in these patients [30]. The tolerability and generalizable response rate is not clear with ERK inhibition; a follow-up study within the NCI-MATCH is currently being conducted with the ERK inhibitor ulixertinib in this same population. Alternative RAF inhibitors could also play a role. So-called “dimer disrupting” or “paradox breaker” RAF inhibitors have also shown preliminary activity; these agents block MAPK signaling by disrupting BRAF-containing dimers, while also blocking monomeric BRAF signaling [31, 32]. This theoretically would facilitate broad targeting of MAPK-driven tumors, including *BRAF* V600, *BRAF* non-V600, and *RAS* mutant tumors. This strategy also prevents paradoxical MAPK upregulation in *BRAF* wild type cells, thus avoiding the cutaneous squamous cell cancers that are promoted by mutant specific BRAF inhibitors [33]. However, further development of this agent (PLX8394) is not clear (NCT02428712).

In conclusion, the activity of MEK inhibition (specifically, trametinib) outside the narrow window of patients with melanoma and class 2 mutations, does not appear robust in cancers harboring non-V600 *BRAF* mutations. Innovative approaches to block parallel signaling networks or more thorough downstream MAPK pathway activation may hold promise for this challenging population.

## References

[1] Long GV , Eroglu Z , Infante J , Patel S , Daud A , Johnson DB , Gonzalez R , Kefford R , Hamid O , Schuchter L , Cebon J , Sharfman W , McWilliams R , et al. Long-Term Outcomes in Patients With BRAF V600-Mutant Metastatic Melanoma Who Received Dabrafenib Combined With Trametinib. J Clin Oncol. 2018; 36:667–673. 10.1200/JCO.2017.74.1025. 28991513 PMC10466457

[2] Salama AKS , Li S , Macrae ER , Park JI , Mitchell EP , Zwiebel JA , Chen HX , Gray RJ , McShane LM , Rubinstein LV , Patton D , Williams PM , Hamilton SR , et al. Dabrafenib and Trametinib in Patients With Tumors With BRAF(V600E) Mutations: Results of the NCI-MATCH Trial Subprotocol H. J Clin Oncol. 2020; JCO2000762. 10.1200/JCO.20.00762. 32758030 PMC7676884

[3] Diamond EL , Durham BH , Ulaner GA , Drill E , Buthorn J , Ki M , Bitner L , Cho H , Young RJ , Francis JH , Rampal R , Lacouture M , Brody LA , et al. Efficacy of MEK inhibition in patients with histiocytic neoplasms. Nature. 2019; 567:521–524. 10.1038/s41586-019-1012-y. 30867592 PMC6438729

[4] Planchard D , Besse B , Groen HJM , Souquet PJ , Quoix E , Baik CS , Barlesi F , Kim TM , Mazieres J , Novello S , Rigas JR , Upalawanna A , D'Amelio AM Jr , et al. Dabrafenib plus trametinib in patients with previously treated BRAF(V600E)-mutant metastatic non-small cell lung cancer: an open-label, multicentre phase 2 trial. Lancet Oncol. 2016; 17:984–993. 10.1016/S1470-2045(16)30146-2. 27283860 PMC4993103

[5] Tiacci E , Park JH , De Carolis L , Chung SS , Broccoli A , Scott S , Zaja F , Devlin S , Pulsoni A , Chung YR , Cimminiello M , Kim E , Rossi D , et al. Targeting Mutant BRAF in Relapsed or Refractory Hairy-Cell Leukemia. N Engl J Med. 2015; 373:1733–1747. 10.1056/NEJMoa1506583. 26352686 PMC4811324

[6] Gao J , Aksoy BA , Dogrusoz U , Dresdner G , Gross B , Sumer SO , Sun Y , Jacobsen A , Sinha R , Larsson E , Cerami E , Sander C , Schultz N . Integrative analysis of complex cancer genomics and clinical profiles using the cBioPortal. Sci Signal. 2013; 6:pl1. 10.1126/scisignal.2004088. 23550210 PMC4160307

[7] Zehir A , Benayed R , Shah RH , Syed A , Middha S , Kim HR , Srinivasan P , Gao J , Chakravarty D , Devlin SM , Hellmann MD , Barron DA , Schram AM , et al. Mutational landscape of metastatic cancer revealed from prospective clinical sequencing of 10,000 patients. Nat Med. 2017; 23:703–713. 10.1038/nm.4333. 28481359 PMC5461196

[8] AACR Project GENIE Consortium. AACR Project GENIE: Powering Precision Medicine through an International Consortium. Cancer Discov. 2017; 7:818–831. 10.1158/2159-8290.CD-17-0151. 28572459 PMC5611790

[9] Wan PT , Garnett MJ , Roe SM , Lee S , Niculescu-Duvaz D , Good VM , Jones CM , Marshall CJ , Springer CJ , Barford D , Marais R . Mechanism of activation of the RAF-ERK signaling pathway by oncogenic mutations of B-RAF. Cell. 2004; 116:855–867. 10.1016/S0092-8674(04)00215-6. 15035987

[10] Yao Z , Yaeger R , Rodrik-Outmezguine VS , Tao A , Torres NM , Chang MT , Drosten M , Zhao H , Cecchi F , Hembrough T , Michels J , Baumert H , Miles L , et al. Tumours with class 3 BRAF mutants are sensitive to the inhibition of activated RAS. Nature. 2017; 548:234–238. 10.1038/nature23291. 28783719 PMC5648058

[11] Yao Z , Torres NM , Tao A , Gao Y , Luo L , Li Q , de Stanchina E , Abdel-Wahab O , Solit DB , Poulikakos PI , Rosen N . BRAF Mutants Evade ERK-Dependent Feedback by Different Mechanisms that Determine Their Sensitivity to Pharmacologic Inhibition. Cancer Cell. 2015; 28:370–383. 10.1016/j.ccell.2015.08.001. 26343582 PMC4894664

[12] Dahlman KB , Xia J , Hutchinson K , Ng C , Hucks D , Jia P , Atefi M , Su Z , Branch S , Lyle PL , Hicks DJ , Bozon V , Glaspy JA , et al. BRAFL597 Mutations in Melanoma Are Associated with Sensitivity to MEK Inhibitors. Cancer Discov. 2012; 2:791–797. 10.1158/2159-8290.CD-12-0097. 22798288 PMC3449158

[13] Hutchinson KE , Lipson D , Stephens PJ , Otto G , Lehmann BD , Lyle PL , Vnencak-Jones CL , Ross JS , Pietenpol JA , Sosman JA , Puzanov I , Miller VA , Pao W . BRAF Fusions Define a Distinct Molecular Subset of Melanomas with Potential Sensitivity to MEK Inhibition. Clin Cancer Res. 2013; 19:6696–6702. 10.1158/1078-0432.CCR-13-1746. 24345920 PMC3880773

[14] Dankner M , Lajoie M , Moldoveanu D , Nguyen TT , Savage P , Rajkumar S , Huang X , Lvova M , Protopopov A , Vuzman D , Hogg D , Park M , Guiot MC , et al. Dual MAPK Inhibition Is an Effective Therapeutic Strategy for a Subset of Class II BRAF Mutant Melanomas. Clin Cancer Res. 2018; 24:6483–6494. 10.1158/1078-0432.CCR-17-3384. 29903896

[15] Kim KB , Kefford R , Pavlick AC , Infante JR , Ribas A , Sosman JA , Fecher LA , Millward M , McArthur GA , Hwu P , Gonzalez R , Ott PA , Long GV , et al. Phase II study of the MEK1/MEK2 inhibitor Trametinib in patients with metastatic BRAF-mutant cutaneous melanoma previously treated with or without a BRAF inhibitor. J Clin Oncol. 2013; 31:482–489. 10.1200/JCO.2012.43.5966. 23248257 PMC4878037

[16] Marconcini R , Galli L , Antonuzzo A , Bursi S , Roncella C , Fontanini G , Sensi E , Falcone A . Metastatic BRAF K601E-mutated melanoma reaches complete response to MEK inhibitor trametinib administered for over 36 months. Exp Hematol Oncol. 2017; 6:6. 10.1186/s40164-017-0067-4. 28344857 PMC5361706

[17] Johnson DB , Zhao F , Noel M , Riely GJ , Mitchell EP , Wright JJ , Chen HX , Gray RJ , Li S , McShane LM , Rubinstein LV , Patton D , Williams PM , et al. Trametinib Activity in Patients with Solid Tumors and Lymphomas Harboring BRAF Non-V600 Mutations or Fusions: Results from NCI-MATCH (EAY131). Clin Cancer Res. 2020; 26:1812–1819. 10.1158/1078-0432.CCR-19-3443. 31924734 PMC7165046

[18] Corcoran RB , Atreya CE , Falchook GS , Kwak EL , Ryan DP , Bendell JC , Hamid O , Messersmith WA , Daud A , Kurzrock R , Pierobon M , Sun P , Cunningham E , et al. Combined BRAF and MEK Inhibition With Dabrafenib and Trametinib in BRAF V600-Mutant Colorectal Cancer. J Clin Oncol. 2015; 33:4023–4031. 10.1200/JCO.2015.63.2471. 26392102 PMC4669588

[19] Subbiah V , Puzanov I , Blay JY , Chau I , Lockhart AC , Raje NS , Wolf J , Baselga J , Meric-Bernstam F , Roszik J , Diamond EL , Riely GJ , Sherman EJ , et al. Pan-Cancer Efficacy of Vemurafenib in BRAF (V600)-Mutant Non-Melanoma Cancers. Cancer Discov. 2020; 10:657–663. 10.1158/2159-8290.CD-19-1265. 32029534 PMC7196502

[20] Ross JS , Wang K , Chmielecki J , Gay L , Johnson A , Chudnovsky J , Yelensky R , Lipson D , Ali SM , Elvin JA , Vergilio JA , Roels S , Miller VA , et al. The distribution of BRAF gene fusions in solid tumors and response to targeted therapy. Int J Cancer. 2016; 138:881–890. 10.1002/ijc.29825. 26314551 PMC5049644

[21] Jain P , Silva A , Han HJ , Lang SS , Zhu Y , Boucher K , Smith TE , Vakil A , Diviney P , Choudhari N , Raman P , Busch CM , Delaney T , et al. Overcoming resistance to single-agent therapy for oncogenic BRAF gene fusions via combinatorial targeting of MAPK and PI3K/mTOR signaling pathways. Oncotarget. 2017; 8:84697–84713. 10.18632/oncotarget.20949. 29156677 PMC5689567

[22] Johnson DB , Dahlman KB . Class Matters: Sensitivity of BRAF-Mutant Melanoma to MAPK Inhibition. Clin Cancer Res. 2018; 24:6107–6109. 10.1158/1078-0432.CCR-18-1795. 30042206 PMC6295213

[23] Coit DG , Thompson JA , Albertini MR , Barker C , Carson WE , Contreras C , Daniels GA , DiMaio D , Fields RC , Fleming MD , Freeman M , Galan A , Gastman B , et al. Cutaneous Melanoma, Version 2.2019, NCCN Clinical Practice Guidelines in Oncology. J Natl Compr Canc Netw. 2019; 17:367–402. 10.6004/jnccn.2019.0018. 30959471

[24] Negrao MV , Raymond VM , Lanman RB , Robichaux JP , He J , Nilsson MB , Ng PKS , Amador BE , Roarty EB , Nagy RJ , Banks KC , Zhu VW , Ng C , et al. Molecular Landscape of BRAF-Mutant NSCLC Reveals an Association Between Clonality and Driver Mutations and Identifies Targetable Non-V600 Driver Mutations. J Thorac Oncol. 2020; 15:1611–1623. 10.1016/j.jtho.2020.05.021. 32540409 PMC7529990

[25] Menzer C , Menzies AM , Carlino MS , Reijers I , Groen EJ , Eigentler T , de Groot JWB , van der Veldt AAM , Johnson DB , Meiss F , Schlaak M , Schilling B , Westgeest HM , et al. Targeted Therapy in Advanced Melanoma With Rare BRAF Mutations. J Clin Oncol. 2019; 37:3142–3151. 10.1200/JCO.19.00489. 31580757 PMC10448865

[26] Arend RC , Davis AM , Chimiczewski P , O'Malley DM , Provencher D , Vergote I , Ghamande S , Birrer MJ . EMR 20006-012: A phase II randomized double-blind placebo controlled trial comparing the combination of pimasertib (MEK inhibitor) with SAR245409 (PI3K inhibitor) to pimasertib alone in patients with previously treated unresectable borderline or low grade ovarian cancer. Gynecol Oncol. 2020; 156:301–307. 10.1016/j.ygyno.2019.12.002. 31870556

[27] Ragon BK , Odenike O , Baer MR , Stock W , Borthakur G , Patel K , Han L , Chen H , Ma H , Joseph L , Zhao Y , Baggerly K , Konopleva M , et al. Oral MEK 1/2 Inhibitor Trametinib in Combination With AKT Inhibitor GSK2141795 in Patients With Acute Myeloid Leukemia With RAS Mutations: A Phase II Study. Clin Lymphoma Myeloma Leuk. 2019; 19:431–440.e13. 10.1016/j.clml.2019.03.015. 31056348 PMC6626580

[28] Aasen SN , Parajuli H , Hoang T , Feng Z , Stokke K , Wang J , Roy K , Bjerkvig R , Knappskog S , Thorsen F . Effective Treatment of Metastatic Melanoma by Combining MAPK and PI3K Signaling Pathway Inhibitors. Int J Mol Sci. 2019; 20:4235. 10.3390/ijms20174235. 31470659 PMC6747502

[29] Kopetz S , Grothey A , Yaeger R , Van Cutsem E , Desai J , Yoshino T , Wasan H , Ciardiello F , Loupakis F , Hong YS , Steeghs N , Guren TK , Arkenau HT , et al. Encorafenib, Binimetinib, and Cetuximab in BRAF V600E-Mutated Colorectal Cancer. N Engl J Med. 2019; 381:1632–1643. 10.1056/NEJMoa1908075. 31566309

[30] Sullivan RJ , Infante JR , Janku F , Wong DJL , Sosman JA , Keedy V , Patel MR , Shapiro GI , Mier JW , Tolcher AW , Wang-Gillam A , Sznol M , Flaherty K , et al. First-in-Class ERK1/2 Inhibitor Ulixertinib (BVD-523) in Patients with MAPK Mutant Advanced Solid Tumors: Results of a Phase I Dose-Escalation and Expansion Study. Cancer Discov. 2018; 8:184–195. 10.1158/2159-8290.CD-17-1119. 29247021

[31] Yao Z , Gao Y , Su W , Yaeger R , Tao J , Na N , Zhang Y , Zhang C , Rymar A , Tao A , Timaul NM , McGriskin R , Outmezguine NA , et al. RAF inhibitor PLX8394 selectively disrupts BRAF dimers and RAS-independent BRAF-mutant-driven signaling. Nat Med. 2019; 25:284–291. 10.1038/s41591-018-0274-5. 30559419 PMC6404779

[32] Gunderwala AY , Nimbvikar AA , Cope NJ , Li Z , Wang Z . Development of Allosteric BRAF Peptide Inhibitors Targeting the Dimer Interface of BRAF. ACS Chem Biol. 2019; 14:1471–1480. 10.1021/acschembio.9b00191. 31243962 PMC6733264

[33] Su F , Viros A , Milagre C , Trunzer K , Bollag G , Spleiss O , Reis-Filho JS , Kong X , Koya RC , Flaherty KT , Chapman PB , Kim MJ , Hayward R , et al. RAS mutations in cutaneous squamous-cell carcinomas in patients treated with BRAF inhibitors. N Engl J Med. 2012; 366:207–215. 10.1056/NEJMoa1105358. 22256804 PMC3724537

